# Assessment of Corrosion Performance of Steel Rebar in Snail Shell Ash Blended Cements under Marine Environments

**DOI:** 10.3390/ma14237286

**Published:** 2021-11-28

**Authors:** Muralidharan Raghav, Subbiah Karthick, Taejoon Park, Han-Seung Lee

**Affiliations:** 1 Department of Civil Engineering, PSG Institute of Technology and Applied Research, Neelambur, Coimbatore 641062, India; raghav09psgitech@gmail.com; 2 Department of Architectural Engineering, Hanyang University, 55 Hanyangdaehak-ro, Sangrok-gu, Ansan-si 15588, Gyeonggi-do, Korea; 3 Department of Robotics Engineering, Hanyang University, 55 Hanyangdaehak-ro, Ansan-si 15588, Gyeonggi-do, Korea; taejoon@hanyang.ac.kr

**Keywords:** snail shell ash, reinforcement corrosion, corrosion resistance property, mechanical property

## Abstract

An attempt has been made on a constructive approach to evaluate the performance of snail shell ash (SSA) for its corrosion performance under marine environments. Corrosion performance of steel rebar in chloride-contaminated SSA with (0% to 50%) replacement levels of cement extract medium was examined through electrochemical and weight loss techniques. Initially, snail shell powder (SSP) is made by pulverizing and subsequently SSA is by thermal decomposition methods. Both SSP and SSA were characterized by X-ray diffraction, Fourier transforms infrared spectroscopy, scanning electron microscopy, and energy dispersion X-ray spectroscopy. Concrete cubes with 0% to 50% replacement levels of cement by SSA were evaluated for their mechanical properties. A critical level of 20 to 30% SSA improved both corrosion resistance and strength of concrete. Extrapolation modeling for the strength and corrosion rate with respect to later age were made. SSA is a suitable replacement material for natural limestone in cement productions.

## 1. Introduction

Concrete structures have played a predominant role in the modernization of developing countries and cement is an essential material in construction industries. Ordinary Portland cement (OPC) is one of the essential ingredients for concrete production, which is one of the second most-used material in the world next to water [1]. With every country increasing infrastructure for global urbanization, this has increased the demand of cement within the construction industry every day. Worldwide cement production is approximately 4.4 million metric tons in 2020 [2], which is set to increase by 12–23% by 2050 from the present level [3]. Commonly, 1.5 to 1.8 tonnes of natural limestone and 0.4 tonnes of clay were used for each tone of cement production [4], reducing the natural resource and also around 5–8% of CO_2_ was emitted from the cement industry [1,5], which leads to an environmental problem. Furthermore, the current consumption of natural resources could create a problem that will affect the entire cement industry due to the scarcity of natural resources in the future. In addition, the restrain of industrial, agriculture, and animal food waste is one of the major problems to maintain a clean environment in every country. Therefore, considering environmental sustainability and life of future generations, the cement industry is reducing the consumption of natural limestone by using various industrial and agricultural waste materials (supplementary cementitious materials) as partially reducing the cement content in construction [6]. This might prevent the depletion of natural resources and maintain the ecological balance. For example, industrial byproducts like fly ash (FA) [7,8,9], granulated blast furnace slag (GGBS) [10,11], silica fume [12], and agricultural waste rice husk ash (RHA) [13,14], sugarcane bagasse ash (SBA) [15], and palm oil ash [16] were used as a partial replacement of cement in the construction of concrete structures, which will reduce the consumption of natural limestone for cement production and helps to maintain the natural resources.

One such food waste is snail shell (SS), which is obtained from a coastal area; people consume the edible part (50%) as seafood and the remaining 50% of the shell is disposed of as a waste product, with the seafood industry producing approximately 45.3 million kg of snail shell waste [17], which is finally disposed to landfills. The chemical composition of snail shells is greater than 90% of calcium carbonate (CaCO_3_), which is nearly equal to limestone powder [18]. A considerable amount of research has been reported that the reuse of SS waste was used in biomedical [19], catalyst [20], wastewater purification [21], removal of heavy metals in wastewater [22], and construction materials [23,24,25]. Lertwattanaruk et al. made cement mortar with 5%, 10%, 15%, and 20% SSP as partial replacements of cement and they conclude that the cement mortar with snail shell increased setting time, lower drying shrinkage, decrease in thermal conductivity, and good compressive strength [18]. Nkrumah and Dankwah studied the blending of rice husk ash and snail shells as partial replacements for Portland cement for making blocks [26]. Snail shell was used as a fine aggregate at various replacement levels from 20% to 100% and the result was compared with river sand [27]. Adeala and Olaoye studied the strength properties of snail shell ash (SSA) produced after the heat treatment of African giant snail shells with various replacement levels of cement [28]. The measured compressive strength values were 25.17 N/mm^2^, 23.92 N/mm^2^, and 24.63 N/mm^2^ at 0% (control), 15%, and 20% replacement levels of cement with SSA, respectively. The better physical and mechanical properties were observed up to a 20% replacement level of OPC. Snail shell, clamshell, and the snail-clam mixture was tried as a partial replacement of cement in construction. The maximum partial replacement by weight of cement with SSA is 20% with compressive strength at 20 N/mm^2^, clamshell ash at 25% with compressive strength at 30 N/mm^2^, and snail-clam shell ash mix at 25% with compressive strength at 40 N/mm^2^ [29]. The snail shell contains a large amount of CaCO_3_ which may be considered as an alternative material for cement production instead of natural limestone [18]. The heat treatment of snail shells at 1000 °C form snail shell ash (SSA), which mostly contains CaO. The major compounds of CaCO_3_ (SS) are decomposed to form CaO (SSA) at a temperature of 1000 °C. The SSA enriched with CaO is a major constituent in Portland cement, which is considered a sustainable binder material in concrete construction. The utilization of SSA reduces the consumption of OPC in concrete construction, which leads to prevent the depletion of natural resources and also reduce environmental pollution by utilizing the large tons of landfill waste snail shells. A considerable number of studies have focused on the improvement of physical and mechanical properties of concrete but systematic and detailed studies on the corrosion performance of steel rebar in cement replaced by SSA are rare. Therefore, the identification of optimum replacement level of cement with SSA is important for concrete preparation without affecting traditional concrete properties and a realistic assessment of the corrosion performance of reinforced steel rebar in SSA-blended concrete.

The objective of the present investigation is to study the influence of SSA on the corrosion resistance of steel rebar and the strength of concrete. In this direction, this paper is divided into three parts. The first part details the preparation and characterization of SSA from raw snail shell, the second part is to evaluate the corrosion performance of steel rebar in chloride contaminated cement containing various replacement levels of SSA extracts (0, 10, 20, 30, 40, and 50% of SSA), which is examined by electrochemical methods and gravimetric weight loss methods. The corroded surfaces are examined using XRD and SEM-EDS. The third part is to study the mechanical properties of concrete with different replacement levels of SSA (0, 10, 20, 30, 40, and 50%).

## 2. Materials and Methods

### 2.1. Materials Used

In the present investigation, ordinary Portland cement (OPC) and thermomechanical treated (TMT) steel rebars were used. OPC used is as per ASTM C150 [30]. The chemical compositions of the OPC and TMT steel rebar are given in Table 1. The local clean river sand (fineness modulus of medium sand equal to 2.6) was used as fine aggregate and locally available well-graded aggregates (blue metal jelly) of a normal size greater than 12 mm and less than 16 mm was used as a coarse aggregate. The fineness modulus of fine and coarse aggregates used were 2.75 and 7.25, respectively. The specific gravity of fine and coarse aggregates is 2.69 and 2.85, respectively. Water absorption of fine and coarse aggregates are 0.5% and 0.1%, respectively.

### 2.2. Preparation and Characterization of SSA

#### 2.2.1. Preparation Methodology of SSA from Raw Snail Shell

**Figure 1:**
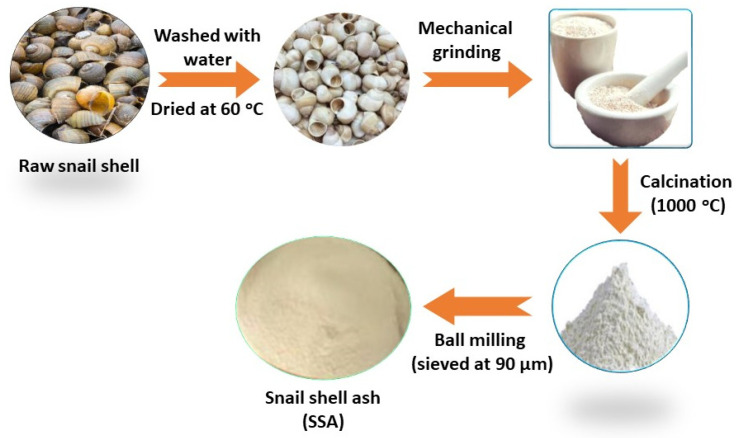
Flow chart for the preparation methodology of snail shell ash (SSA).

Raw snail shells were collected from a local sea area in Tamil Nadu, India. The snail shells were cleaned in tap water several times to remove organic and other foreign matters. Finally, the washed snail shells were immersed in acetone and washed with distilled water, thereafter dried at 60 ± 3 °C. The snail shell must be free from chlorides and sulphates, since the aggressive anions that accelerate the corrosion of reinforced concrete structures. After drying, the snail shell was mechanically crushed into fine pieces, which is called SSP. Then, the SSP was calcined at the muffle furnace at 1000 ± 20 °C for 3 h. After calcination, the obtained snail shell ash (SSA) was pulverized and obtained fine SSA sieving through a 90-micron sieve. The preparation methodology of SSA is given in Figure 1.

#### 2.2.2. Characterization of SSA

The crystal phase structure of the SSP and SSA were characterized using X-ray diffraction spectroscopy. A computer-controlled XRD system, D/MAX-2500, Rigaku, Japan with CuαK radiation (λ = 1.54059 Å) generated at 40 kV and 20 A, was used for recording XRD patterns. The identification of diffraction peaks was performed by employing ‘peak search’ and ‘search match’ programs in the software (version 2.0, PA Analytical, X’pert High score plus).

SSP and SSA were subjected to Fourier transform infrared spectroscopy (FTIR) (FT-IR, Perkin Elmer UATR Two, Akron, OH, USA) spectroscopy and the FTIR spectrum was recorded in the transmittance mode in the range of 4000–400 cm^−1^.

The surface morphology and elemental compositions of SSP and SSA were characterized by scanning electron microscopy (SEM, MIRA3, TESCAN, Brno, Czech Republic) and energy-dispersive X-ray spectroscopy (EDS) respectively.

### 2.3. Preparation of the Cement Extracts Using SSA

The cement extracts were prepared at different replacement levels of OPC by SSA namely 0%, 10%, 20, 30, 40, and 50%. For example, the O-SSA10 cement extract solution was prepared by 90 g of OPC + 10% of SSA (i.e., cement replaced with 10% of SSA), then 100 mL of distilled water was added to the above mixture and shaken vigorously using a mechanical shaker for 2 h. After that, the undissolved cement particles were removed by the filtration (Whatman filter paper) method. The detailed formulation of cement extracts with different replacement levels of SSA is given in Table 2.

For comparison, the cement extract was also prepared without SSA via a similar procedure. All the cement extracts were subsequently contaminated with 3.5% NaCl to represent the marine environment. The alkalinity (pH) of the various cement extracts were measured by a digital portable pH meter accuracy of ±0.01 and the results are given in Table 2.

### 2.4. Preparation of Steel Rebar

TMT rebar with a 1.2 cm diameter and 10 cm in length were used for this study. The oxide layer of the steel rebar was removed by immersing it in pickling acid (as per ASTM G1-03) [31] for 3–5 min. After that, the steel rebar was washed with water, degreased with acetone, and then dried at 60 ± 3 °C. The 15.072 cm^2^ area of the steel rebar was selected as a working area for corrosion studies and the other portion of the steel rebar was sealed with epoxy.

### 2.5. Evaluation of Corrosion Performance of Steel Rebars in Various Cement Extracts

The prepared steel rebars were immersed in chloride-contaminated O-SSA0, O-SSA10, O-SSA20, O-SSA30, O-SSA40, and O-SSA50 cement extract solutions as per the ASTM G31-12a [32] standard procedure for laboratory corrosion testing of metals. The corrosion behavior of steel rebar immersed in O-SSA0, O-SSA10, O-SSA20, O-SSA30, O-SSA40, and O-SSA50 cement extract solutions was evaluated by electrochemical methods as well as a gravimetric weight loss method.

#### 2.5.1. Electrochemical Methods

The corrosion behavior of the steel rebar immersed in chloride contaminated O-SSA0, O-SSA10, O-SSA20, O-SSA30, O-SSA40, and O-SSA50 cement extracts solution were monitored by chrono-potential studies. The open circuit potential (OCP) of the steel rebars was measured with respect to saturated calomel electrode (SCE) using a digital very high impedance multimeter for an exposure period of 30 days. The OCP measurement of the steel rebar was done according to the ASTM C876-15 [33].

The potentiostat (VersaSTAT Princeton Applied Research, Oak Ridge, TN, USA) was used for carried out potentiodynamic polarization and electrochemical impedance spectroscopy (EIS) experiments using a conventional three-electrode cell assembly. Duplicate experiments were carried out for electrochemical studies. The stainless steel and SCE were used as a counter electrode and a reference electrode, respectively. The steel rebar (15.072 cm^2^) was used as a working electrode. The chloride contaminated O-SSA0, O-SSA10, O-SSA20, O-SSA30, O-SSA40, and O-SSA50 cement extract solutions were used as an electrolyte. The potentiodynamic polarization of steel rebars was carried out by applying the potential ranges from −200 mV to +200 mV from the OCP of the steel rebar and the potential scan rate was 0.1 mV/s. The EIS measurements were carried out at the OCP of steel rebars by applying an AC signal 10-mV amplitude in frequency ranges from 100 kHz to 0.01 Hz. The impedance parameters and corrosion kinetic parameters were evaluated by fitting the experimental data in the constant phase element and Tafel region. The system in-built software of Metrohm Autolab Nova 1.10 (VersaSTAT (Princeton applied Research, Oak Ridge, TN, USA)) was used for curve fitting analysis. The Tafel plots, Nyquist plots, and Bode plots were recorded at different exposure periods namely 1 day (initial), 15 days, and 30 days at a room temperature of 25 ± 3 °C.

#### 2.5.2. Gravimetric Weight Loss Method

The gravimetric weight loss measurements were performed as per the ASTM standard. The surface cleaned steel rebars were taken for this study. The initial weight and dimension of the steel rebars were noted, then the steel rebars were immersed in chloride contaminated OSSA, OSSA10, OSSA20, OSSA30, OSSA40, and OSSA50 cement extract solutions (ASTM G31-12a) [32] for an exposure period of 30 days. After 30 days of exposure, the steel rebars were taken and visually examined for any corrosion spots on the steel surface. Subsequently, the formation of corrosion products on the steel rebar surface was cleaned by acid pickling (ASTM G1-03) [32] and washed with distilled water, degreased with acetone, and then dried at 60 ± 3 °C. The initial and final weight of the steel rebars were recorded using the Mettler Toledo balance (Torunska 5, 26-600, Radom, Poland) and accuracy of 0.0001 g. The corrosion rate of the steel rebar immersed in chloride contaminated OSSA, OSSA10, OSSA20, OSSA30, OSSA40, and OSSA50 cement extract solutions were determined using the equation as follows [34]:

(1) $$\mathrm{Corrosion}\;\mathrm{rate}=\frac{87.6\;\left(W_{i}-W_{f}\right)}{\mathrm{DAt}}$$

where W_f_ and W_i_ are the final and initial weight of the steel rebar (mg):

‘D’ is the density of the steel rebar (g/cm^3^);

‘A’ denotes the exposure area of the steel rebar (cm^2^);

‘t’ represents the exposure time (h).

### 2.6. Characterization of Corrosion Products

After an exposure period of 30 days, the steel rebars were taken and the formation of corrosion products were examined and rinsed with distilled water then wiped with tissue paper. After that, the formation of corrosion products on the steel rebar surface was characterized by X-ray diffraction (XRD), scanning electron microscope (SEM), coupled with energy-dispersive X-ray spectroscopy (EDS); (TESCAN MIRA3, Brno, Czech Republic).

### 2.7. Casting of Concrete Specimens

The mix ratio of cement, fine, and coarse aggregates were 1:1.71:3.1 respectively, with a 0.5 water to cement (w/c) ratio was used. The size of the concrete cubes (150 mm × 150 mm × 150 mm) and cylinders (15 mm dia × 100 mm length) were cast with different replacements level of cement using SSA (10% to 50%). The detailed concrete mix proportion used for casting concrete specimens is given in Table 3. During casting, the concrete specimen molds were adequately compacted using a table vibrator to avoid the voids. After 24 h, the concrete specimens were demolded and subjected to water curing for 28 days.

#### Evaluation of Mechanical Properties

The compressive and split tensile strengths of the concrete were performed as per ASTM: C39/C39M-21 [35] and ASTM:C496/C496M-17 [36], respectively. After 28 days of curing, the compressive and split tensile strength tests were carried out for the concrete specimens using a compression testing machine of 2000 kN capacity. The test was carried out on triplicate specimens and average compressive and split tensile strength values were noted. The compressive and split tensile strength was calculated from the following equations:

For compressive strength:

(2) $$F_{\mathrm{cs}}\;(N/{\mathrm{mm}}^{2})=\frac{f}{a}$$

where f = ultimate load/force (N); a is the area of the concrete specimen (mm^2^).

For split tensile strength:

(3) $$F_{\mathrm{sp}}\;(N/{\mathrm{mm}}^{2})=\frac{2P}{\pi \mathrm{dl}}$$

where P = failure load (N); d and l are the diameter and length of the concrete specimen (mm).

## 3. Results and Discussion

### 3.1. Characterization of SSP and SSA

#### 3.1.1. X-ray Diffraction

**Figure 2:**
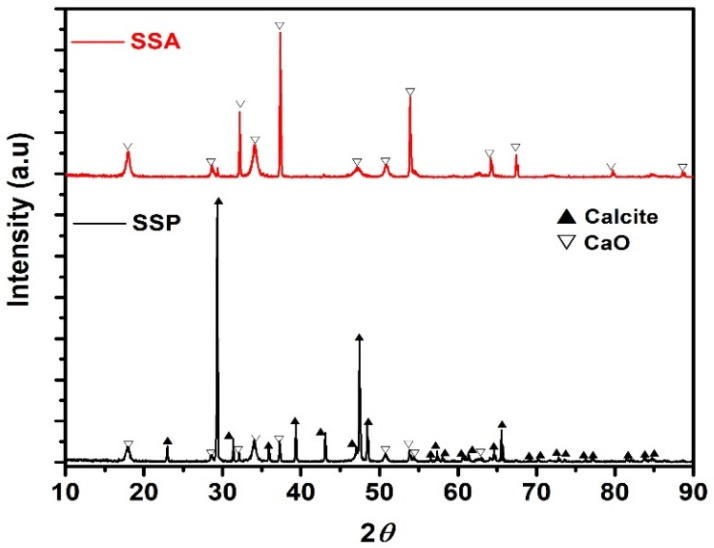
XRD pattern of SSP and SSA.

Figure 2 shows the XRD pattern of SSP and SSA. The XRD pattern of SSP (before calcination) shows the high-intensity crystal plane of calcite (CaCO_3_) diffraction peaks. The diffraction peaks observed at 22.8°, 29.31°, 31.26°, 35.92°, 39.28°, 43.20°, 47.41°, 48.50°, and 65.63° corresponds to the hexagonal crystal phase of calcite (JCPDS: 05-0586). Furthermore, the low-intensity diffraction peaks of calcium oxide (CaO) are also observed in Figure 2 (SSP). Indicating the high degree of crystallinity of calcites is a major component in SSP (before calcination) [37]. The XRD pattern of the SSA (after calcination) is given in Figure 2 (SSA). It can be seen from Figure 2 that the diffraction peaks appeared at 18.08°, 28.55°, 32.13°, 34.19°, 37.43°, 46.97°, 50.89°, 53.81°, 63.44°, 67.47°, 79.72°, and 88.58°, which corresponds to the cubic crystal plan of CaO (JCPDS 002-1088). From these results, it was confirmed that the crystalline size of SSA (presence of calcite) was transformed into a CaO crystal phase at 1000 ± 20 °C which is due to the high thermal transition that occurred in the snail shell, hence the decomposition of calcite into CaO [38].

#### 3.1.2. Fourier Transform Infrared Spectroscopy

**Figure 3:**
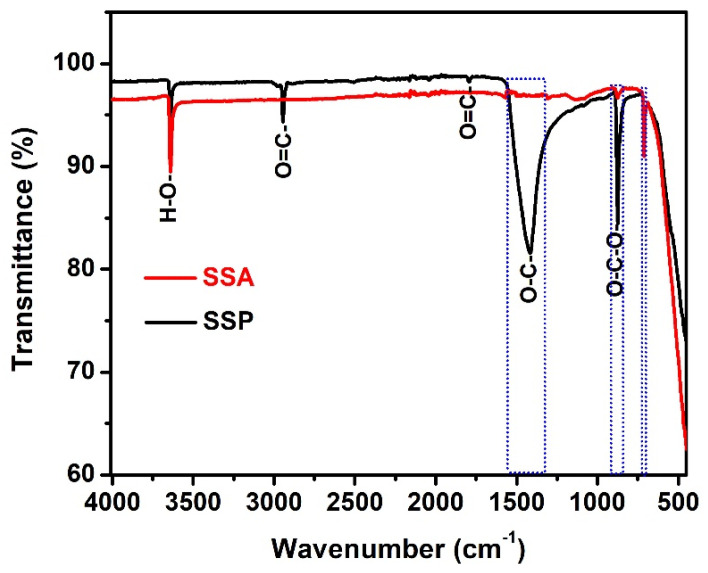
FTIR spectra of SSP and SSA.

The FTIR spectra of the SSP and SSA are shown in Figure 3. From Figure 3 (SSP), the peaks that appeared at 3640 1/cm corresponds to the -OH stretching absorption of hydrogen bonds. The low intensity peaks observed at 2945 cm^−1^ corresponds to the carbonate -C=O group from CaCO_3_. The absorption peaks observed at 1409 cm^−1^ and 875 cm^−1^ attribute to the stretching vibrations bonds of -O-C, and -O-C-O, respectively [20]. It indicates the calcium carbonates (calcite) present in SSP. However, -C=O, -O-C, and -O-C-O peaks have decomposed after calcination of SSA, as shown in Figure 3 (SSA). From these results, it is also confirmed that the presence of calcite in the SSA was decomposed into CaO under 1000 ± 20 °C for 3 h.

#### 3.1.3. Scanning Electron Microscopy/Energy Dispersive X-ray Spectroscopy

**Figure 4:**
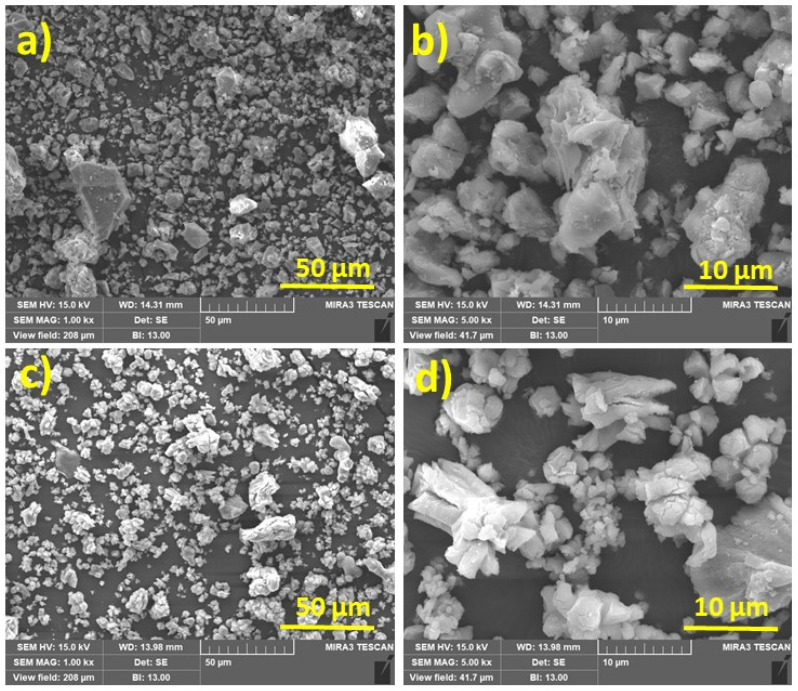
SEM micrographs of SSP (**a**,**b**) and SSA (**c**,**d**).

The surface morphology of SSP and SSA are shown in Figure 4a,b and Figure 4c,d, respectively. The micrographs of SSP in Figure 4a,b illustrate the hexagonal irregular and rough surface texture, which are also agglomerated structures [39]. After calcination of snail shell (SSA), a nearly uniform morphology size of agglomerated semi-cubical like particles and smooth surface were observed from Figure 4c,d. Laskar at al. observed the similar morphology for calcined SSA made from waste snail shell [20].

**Figure 5:**
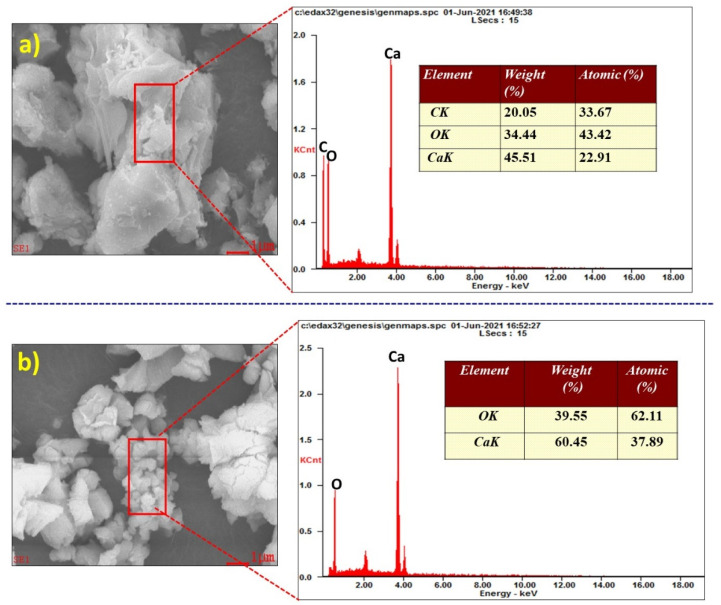
EDS analysis of the SSP (**a**) and SSA (**b**).

Furthermore, the EDS analysis of the SSP and SSA are shown in Figure 5a and Figure 5b, respectively. It can be seen from Figure 5a that the SSP contains major components of calcium (Ca), carbon (C), and oxygen (O). The wt.% of Ca, C, and O were found to be 45.51%, 20.05%, and 34.44%, respectively, indicating the SSP is composed of CaCO_3_. This is in agreement with XRD results as shown in Figure 2 (SSP). Figure 5b shows the EDS analysis of SSA (after calcination) and it can be seen from the figure that higher wt.% of Ca (60.45%) and O (39.55%) in the SSA. These results confirm that the presence of calcite in the SSA was decomposed to form CaO under 1000 ± 20 °C for 3 h. 

### 3.2. Evaluation of Corrosion Performance of Steel Rebars

#### 3.2.1. Electrochemical Techniques

##### Chrono-Potential Studies

**Figure 6:**
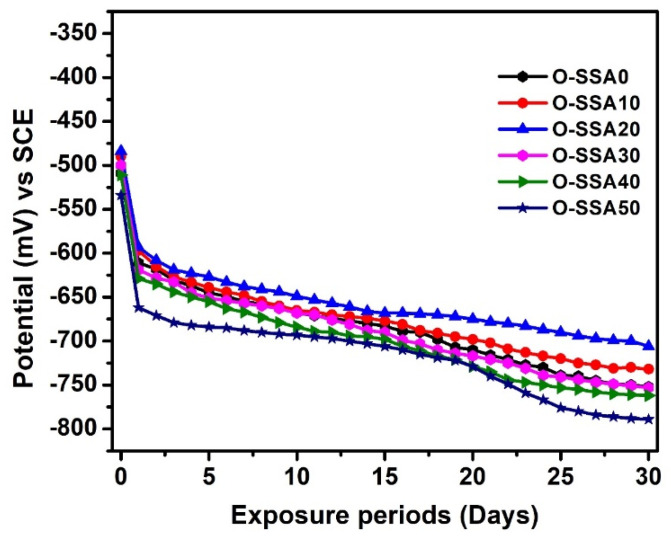
Chrono–potential curves for steel rebars in various cement extracts for an exposure period of 30 days.

Figure 6 relates the chrono-potential curves for steel rebars in various cement extracts for an exposure period of 30 days. It was observed from Figure 6 that the OCP values for the systems, namely O-SSA0, O-SSA10, O-SSA20, O-SSA30, O-SSA40, and O-SSA50 were −611, −597, −593, −619, −629, and −662 mV vs. SCE, respectively for the initial (1 day) exposure. All the systems shifted the OCP towards a more negative direction at the end of 30 days of exposure, indicating the active condition of steel rebars suggest the initiation of corrosion phenomena on the steel rebar/solution interface. However, the steel rebars immersed in the O-SSA20 system showed lesser negative potential when compared to other systems. It was concluded from the chrono-potential studies that the optimum cement replacement level of up to 20% of SSA is sufficiently suitable for better durability of the reinforced concrete structures under marine environments.

##### Potentiodynamic Polarization Studies

**Figure 7:**
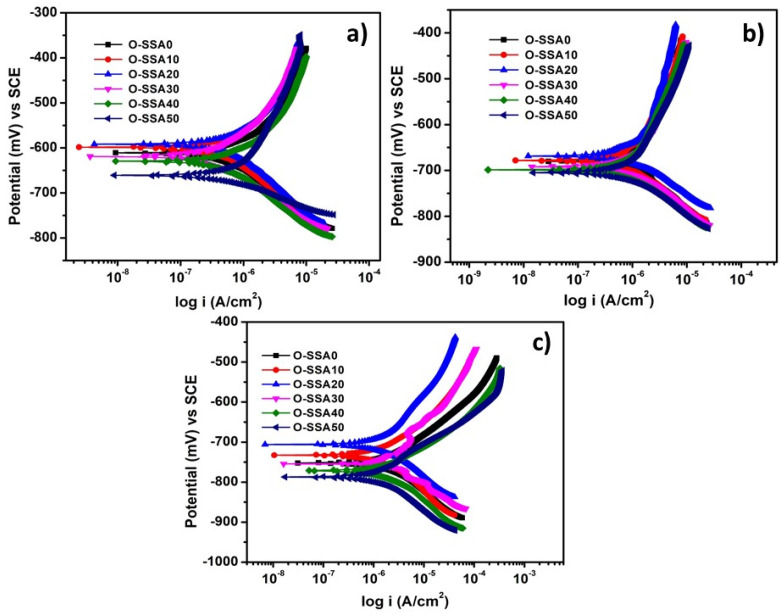
Potentiodynamic polarization curves for steel rebars in various cement extracts for an exposure period of 1 day (**a**); 15 days (**b**); and 30 days (**c**).

Figure 7a–c illustrates the potentiodynamic polarization curves for the steel rebars immersed in chloride contaminated O-SSA0, O-SSA10, O-SSA20, O-SSA30, O-SSA40, and O-SSA50 cement extract solutions for an exposure period of 1, 15, and 30 days. The corresponding corrosion kinetic parameters after fitting of potentiodynamic polarization curves in the Tafel region and fitted data are illustrated in Table 4.

From Figure 7a and Table 4, the corrosion potential (E_corr_) and corrosion current density (I_corr_) values for steel rebars immersed in O-SSA0 were −611 mV and 0.5572 µA/cm^2^ whereas the systems O-SSA10 and O-SSA20 reduced the I_corr_ values by 1.05 times and 1.43 times respectively at 1 day exposure. The significant reduction in I_corr_ values for 10% and 20% replacement levels may be due to the presence of CaO in SSA ionized in water to produce -OH ions and formed a thin portlandite layer on the steel rebar surface [40,41], which initially arrest the chloride attack on the steel rebar surface. On the other hand, further increasing the replacement level of cement up to 30%, 40%, and 50% of SSA, the E_corr_ value of the steel rebars were gradually shifted towards a more negative direction, and its corresponding I_corr_ values were also gradually increased when compared to O-SSA0. For example, system O-SSA50 showed an increasing trend in corrosion rate by 1.57 times compared to control. At a higher replacement level, the larger crowd of hydroxyl ions leads to the poor adherence of surface hydroxyl layers on the steel rebar surface. This will lead to the attack of aggressive anions, such as chloride ions with steel, accelerating the reinforcement corrosion.

The potentiodynamic polarization plots of steel rebars immersed in O-SSA0, O-SSA10, O-SSA20, O-SSA30, O-SSA40, and O-SSA50 after 15 and 30 days of exposure are illustrated in Figure 7b,c respectively. It can be observed from Figure 7b,c and Table 4 that by increasing the exposure periods (15 and 30 days), the E_corr_ values were gradually shifted to a more negative direction and the I_corr_ values are also gradually increased when compared to the initial exposure (1 day). The reduction in the corrosion rate was observed up to 20% replacement levels in both exposure periods (15 and 30 days). For example, the decreasing trend in I_corr_ values for steel rebars immersed in chloride contaminated system O-SSA20 by 1.2 times and 1.51 times for 15 days and 30 days of exposure, respectively. On the other hand, system O-SSA50 showed an increasing trend in I_corr_ values by 1.36 times and 1.79 times for 15 days and 30 days, respectively. Possibly the presence of chloride anions in the solution penetrate the iron hydroxyl layer over the steel rebar surface, thereby weakening the iron hydroxyl layer [42,43]. The trend in the reduction in the corrosion rate for the various systems at the end of 30 days follows the order:

O-SSA20 > O-SSA10 > O-SSA0 > O-SSA30 > O-SSA40 > O-SSA50

2.0969 > 2.7449 > 3.1757 > 3.2715 > 3.7230 > 5.6873 mm/y.

The comparable corrosion rate was obtained up to 20% replacement levels. It was concluded from polarization studies that 20% replacement level of SSA (O-SSA20) showed lesser corrosion current density (0.3857, 1.2536, and 1.7815 μA/cm^2^) and corrosion rate values (0.4539, 1.4755, and 2.0969 mm/y) at all the exposure periods (1, 15, and 30 days), which compared to all other systems.

##### Electrochemical Impedance Spectroscopy

**Figure 8:**
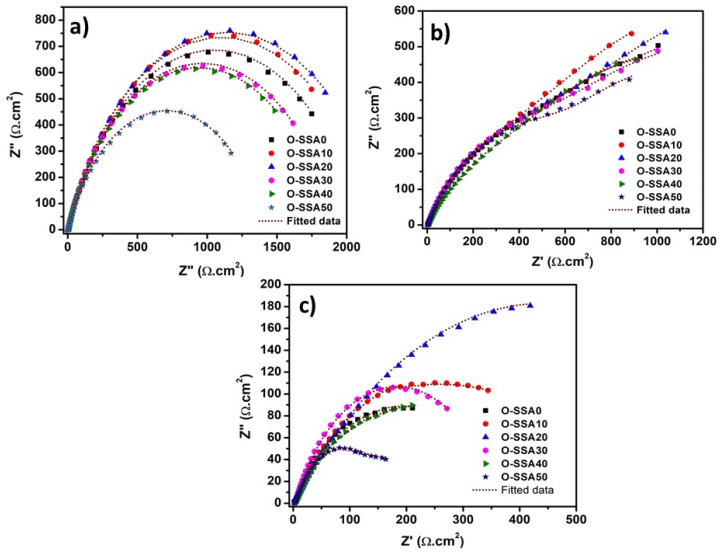
EIS-Nyquist plots for steel rebars in various cement extracts for an exposure period of 1 day (**a**); 15 days (**b**); and 30 days (**c**).

The Nyquist plots of the steel rebar immersed in chloride contaminated O-SSA0, O-SSA10, O-SSA20, O-SSA30, O-SSA40, and O-SSA50 cement extract solutions are shown in Figure 8a–c for the exposure periods of 1, 15, and 30 days. The fitted equivalent circuits are given in Figure S1a,b in the supporting information, and their corresponding impedance parameters are calculated by the fitting of EIS in suitable equivalent circuits and obtained results are given in Table 5. In this equivalent circuit, R_S_ denotes the solution resistance, R_CT_ and CPE_CT_ are the charge transfer resistance and constant phase element of the steel rebars.

Figure 8a and Table 5 illustrate the impedance behavior of steel rebar immersed in chloride contaminated O-SSA0, O-SSA10, O-SSA20, O-SSA30, O-SSA40, and O-SSA50 cement extract solutions at the initial exposure and their corresponding fitted equivalent circuit is shown in Figure S1a. From Table 5, the R_CT_ and CPE_CT_ value of the steel rebars immersed in chloride contaminated O-SSA0, O-SSA10, O-SSA20, O-SSA30, O-SSA40, and O-SSA50 cement extract solutions at 1 day were 2097 Ω·cm^2^ & 0.131 × 10^−2^ Ω^−1^·cm^2^ S^−n^, 2201 Ω·cm^2^ & 0.125 × 10^−2^ Ω^−1^·cm^2^ S^−n^, 2281 Ω·cm^2^ & 0.123 × 10^−2^ Ω^−1^·cm^2^ S^−n^, 1924 Ω·cm^2^ & 0.134 × 10^−2^ Ω^−1^·cm^2^ S^−n^, 1875 Ω·cm^2^ & 0.161 × 10^−2^ Ω^−1^·cm^2^ S^−n^, and 1410 Ω·cm^2^ & 0.164 × 10^−2^ Ω^−1^·cm^2^ S^−n^, respectively. It was observed that the R_CT_ values were increased up to 20% replacement levels when compared to O-SSA0. It indicates the gradual increase of film resistance across the steel-concrete interfacial regions. On the other hand, further increasing the replacement level of cement with 30%, 40%, and 50% of SSA, the R_CT_ values are gradually decreased when compared to O-SSA0. This is due to larger crowding in the solution by the higher concentration of Ca(OH)_2_ (presence of CaO in SSA), which hinders the transport of hydroxyl ions. Hence, the movement of -OH ions towards the steel rebar surface is intricate and thereby prevents the formation of the iron hydroxyl layer, leading to a decrease in the charge transfer resistance values.

At the end of 15 days of exposure, in this equivalent circuit, the R_1_ and CPE_1_ were found, which is shown in Figure 8b and Figure S1b. The following hypothesis is assumed since the steel rebar is exposed in a high alkaline environment, the thin layer of FeOOH adsorbed on the steel rebar surface is the first process and occurs by the following sequences [44,45]:

(4) $${\mathrm{Fe}}^{2+}+{\;\mathrm{OH}}^{-}\;\to \mathrm{FeOH}$$

(5) $$\mathrm{FeOH}+{\mathrm{OH}}^{-}\;\to \mathrm{Fe}{\left(\mathrm{OH}\right)}_{2}+{\;e}^{-}$$

(6) $$Fe{\left(\mathrm{OH}\right)}_{2}+2{\mathrm{Cl}}^{-}\;\to {\mathrm{FeOCl}}_{2}^{-}+H_{2}O$$

(7) $${\mathrm{FeOCl}}_{2}^{-}+H_{2}O\;\to {\mathrm{FeCl}}_{2}+2{\mathrm{OH}}^{-}$$

(8) $${\mathrm{FeCl}}_{2}+H_{2}O\;\to \mathrm{Fe}{\left(\mathrm{OH}\right)}_{2}+2\mathrm{HCl}$$

(9) $$Fe{\left(\mathrm{OH}\right)}_{2}+{\mathrm{OH}}^{-}\;\to \mathrm{FeOOH}+{\;H}_{2}O+{\;e}^{-}.$$

Interestingly, R_1_ and CPE_1_ were found at 15 days of exposure whereas R_1_ and CPE_1_ were absent at the initial exposure. The R_1_ and CPE_1_ denote the charge transfer resistance and constant phase elements of the formation of the iron hydroxyl layer. This may be due to the deficient time for the formation of the iron hydroxyl layer on the steel rebar surface. It can be observed from Figure 8b and Table 5 that the R_1_ values of the steel rebar immersed in chloride contaminated O-SSA0, O-SSA10, O-SSA20, O-SSA30, O-SSA40, and O-SSA50 cement extract solutions were 969.6 Ω·cm^2^, 975.8 Ω·cm^2^, 989.8 Ω·cm^2^, 966.9 Ω·cm^2^, 946.7 Ω·cm^2^, and 920.2 Ω·cm^2^ at the end of 15 days of exposure. Here it is interesting to note that the R_1_ values slightly increased up to 20% of cement replaced by SSA. The presence of CaO in the SSA produce more -OH ions and leads to an increase in the thickness of the iron hydroxyl layer. It has been stated that the formations of FeOOH and α-FeOOH (goethite) on the steel rebar surface were decreased/slow down the rate of corrosion reaction and which is very adherent and stable to reduce weakening the film [45,46]. Hence, the R_1_ values were slightly increased when compared to O-SSA0. However, further increasing the replacement level to 30%, 40%, and 50% of SSA, the R_1_ values were gradually decreased when compared to O-SSA0. The higher concentration of Ca(OH)_2_ (presence of CaO in SSA) in the solution can be encouraged to the crowding of the solution, also limiting the transport of the ions.

Besides, the -OH ions finding their way to the steel rebar surface is difficult, thereby the formation of the iron hydroxyl layer was reduced, hence the charge transfer resistance of the iron hydroxyl layer decreases with increasing the replacement level of cement by 30%, 40%, and 50% of SSA. The R_CT_ value of the steel rebar immersed in chloride contaminated O-SSA0, O-SSA10, O-SSA20, O-SSA30, O-SSA40, and O-SSA50 cement extract solutions were 1063 Ω·cm^2^, 1142 Ω·cm^2^, 1239 Ω·cm^2^, 971 Ω·cm^2^, 894 Ω·cm^2^, and 799 Ω·cm^2^ at the exposure period of 15 days. The R_CT_ values were decreased at 15 days exposure when compared to the initial exposure, which is due to the presence of chloride ions decreasing the coverage of the iron hydroxyl layer, thus increasing the charge transfer between steel rebar and solutions. Hence the R_1_ and R_CT_ values are decreased while increasing the exposure periods.

For example, at the end of 30 days, the Nyquist plots for the steel rebar immersed in chloride contaminated O-SSA0, O-SSA10, O-SSA20, O-SSA30, O-SSA40, and O-SSA50 cement extract solutions are shown in Figure 8c. The R_1_ and R_CT_ values of the steel rebar immersed in chloride contaminated O-SSA0, O-SSA10, O-SSA20, O-SSA30, O-SSA40, and O-SSA50 were 157.9 Ω·cm^2^ & 176 Ω·cm^2^, 180.7 Ω·cm^2^ & 289 Ω·cm^2^, 243.9 Ω·cm^2^ & 385 Ω·cm^2^, 159.5 Ω·cm^2^ & 183 Ω·cm^2^, 157.1 Ω·cm^2^ & 175 Ω·cm^2^, and 100.8 Ω·cm^2^ & 158 Ω·cm^2^ respectively, at a 30-day exposure period. The R_1_ and R_CT_ values of the steel rebar were decreased when compared to 15 days. This might be indicating the presence of chloride ions continuous attack on the iron hydroxyl layer, breaking down the iron hydroxyl layer, thus leading to decreased charge transfer resistance. As a result, the anodic dissolution of the metal was increased by the following sequence [47,48,49]:

(10) $$4\mathrm{FeOOH}+12{\mathrm{Cl}}^{-}\;\to 4{\mathrm{FeCl}}_{3}+2H_{2}O+3O_{2}$$

(11) $$2{\mathrm{FeCl}}_{3}+6H_{2}O\;\to 2\mathrm{Fe}{\left(\mathrm{OH}\right)}_{3}+6{\mathrm{Cl}}^{-}+6H^{+}$$

(12) $$2\mathrm{Fe}{\left(\mathrm{OH}\right)}_{3}\;\to {\mathrm{Fe}}_{2}O_{3}+3H_{2}O\;$$

(13) $${\mathrm{Fe}}^{2+}+2\mathrm{Fe}{\left(\mathrm{OH}\right)}_{3}+\frac{1}{2}O_{2}+2e^{-}\to {\mathrm{Fe}}_{3}O_{4}+3H_{2}O$$

The continuous attack of Cl^−^ ions on the iron hydroxyl layer (FeOOH) to form a soluble FeCl_3_ product, and then FeCl_3_ is further hydrolyzed to form a very porous Fe(OH)_3_ formed on the steel rebar surface. The Fe(OH)_3_ product is not stable and cannot protect the steel rebar against corrosion [48,49] and it is further to form porous Fe_2_O_3_ and Fe_3_O_4_ on the steel rebar surface [50]. Therefore, the R_1_ and R_CT_ values decreased further when increasing exposure periods. Besides, the CPE_CT_ values of the steel rebars were gradually increased due to the increase of the local dielectric constant [51], this might be due to the replacement of -OH ions by chlorides ions on the steel rebar surface while increasing the exposure periods. For example, the CPE_CT_ values of the steel rebar immersed in O-SSA20 were 0.123 × 10^−2^ Ω^−1^·cm^2^ S^−n^, 0.929 × 10^−2^ Ω^−1^·cm^2^ S^−n^, and 1.150 × 10^−2^ Ω^−1^·cm^2^ S^−n^ for the exposure period of 1, 15, and 30 days, respectively. However, these values are slightly lower than OSSA0, which is due to the presence of CaO in the SSA to produce -OH ions and adsorb on the steel surface and reduce the speed of the corrosion reaction [52]. Hence, this result confirms that the optimum replacement level of cement up to 20% SSA is suitable for concrete structures for the betterment of steel rebars.

**Figure 9:**
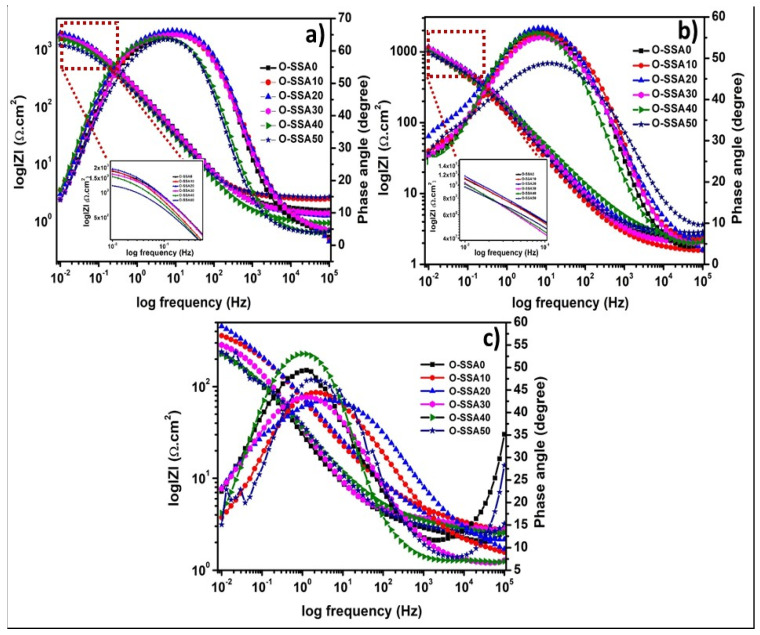
EIS−Bode plots for steel rebars in chloride contaminated various cement extracts for an exposure period of 1 day (**a**); 15 days (**b**); and 30 days (**c**).

The Bode and phase angles plots of the steel rebar immersed in chloride contaminated O-SSA0, O-SSA10, O-SSA20, O-SSA30, O-SSA40, and O-SSA50 cement extracts solution for the exposure period of 1, 15, and 30 days are given in Figure 9a–c, respectively. Figure 9a shows the impedance modulus and phase angle plots for the steel rebar in different replacement levels of SSA in the presence of chloride ions at the exposure period of 1 day. Figure 9a illustrates that the impedance values of the steel rebar immersed in chloride contaminated O-SSA20 showed a slightly higher value at low-frequency regions, which is slightly higher than that of all other systems. The impedance values of the steel rebar in all the systems decreased when increasing the exposure periods (Figure 9a–c). This is might be due to the presence of chloride ions has an impact on the steel rebar surface. Among all, the impedance values of the steel rebar immersed in O-SSA20 showed a higher value at all the exposure periods (1, 15, and 30 days). Figure 9a shows the phase angle plots of the steel rebar immersed in chloride contaminated O-SSA0, O-SSA10, O-SSA20, O-SSA30, O-SSA40, and O-SSA50 cement extracts solution. The broad capacity behavior was observed at the intermediate frequency region (10^2^ to 10^−1^) at the exposure period of 1 day. It indicates the growth of iron hydroxide layer [53] and lower corrosion rate of the steel rebar surface. Further increasing the exposure periods, the phase angle values and capacity behavior as the capacity loop width were decreased at the middle frequency region (10^2^ to 10^−1^). For example, the phase angle values of steel rebars at the middle frequency range (10^2^ to 10^−1^) were around −66.5° to −63.2° for an exposure period of 1 day and around −57.6° to −55° (except O-SSA50) for an exposure period of 15 days (Figure 9b).

It indicates the presence of chloride ions continually attacking the iron hydroxide layer of the steel rebar surface as well as the increased corrosion phenomena on the steel rebar surface. At the exposure periods of 30 days (Figure 9c), the width of the phase angle degree plots was decreased or sharp capacity behavior was observed at the middle frequency range of (10^1^ to 10^−1^) for the steel rebar immersed in the O-SSA0, O-SSA40, and O-SSA30, which may indicate the breakdown of the iron hydroxyl layer and also which means that higher corrosion rate obtained on the steel rebar surface [54]. At the same exposure conditions, the broad capacity behavior observed at the middle frequency regions (10^2^ to 10^−1^) for the steel rebar immersed in the O-SSA10, O-SSA-20, and O-SSA30, which may indicate that the iron hydroxide layer may be present on the steel rear surface and it has become diffused/porous, thus lowering the speed of corrosion rate on the steel rebar surface. This result also confirms that the replacement level of cement with 20% of SSA (O-SSA20) showed better performance at all the exposure periods (1, 15, and 30 days).

#### 3.2.2. Gravimetric Weight Loss Method

At the end of 30 days of exposure, steel rebars were taken from chloride contaminated O-SSA0, O-SSA10, O-SSA20, O-SSA30, O-SSA40, and O-SSA50 cement extracts solution. The corrosion products on the steel rebar surface are visually examined. The photographic image of steel rebars in the chloride contaminated O-SSA0, O-SSA10, O-SSA20, O-SSA30, O-SSA40, and O-SSA50 cement extracts solution are given in Figure S2 in the supporting information. Furthermore, the corrosion products were cleaned using pickling acid (as per ASTM G1-03) [31], then washed with distilled water as well as degreased with acetone and dried at 60 ± 3 °C. The final weight of the rebar was noted and the corrosion rate was calculated as per Equation (1) and given in Table 6. It was observed from Table 6 that the average corrosion rate values of steel rebars immersed in O-SSA0, O-SSA10, O-SSA20, O-SSA30, O-SSA40, and O-SSA50 were 0.7998 mm/y, 0.6913 mm/y, 0.5679 mm/y, 0.7983 mm/y, 0.8586 mm/y, and 0.8869 mm/y, respectively. The steel rebar immersed in O-SSA20 showed a lesser corrosion rate when compared to all other systems. Here, it was concluded that the comparable corrosion rate with control was noticed up to a 30% replacement level. Hence, these results confirm the fact that a cement replacement level of 30% by SSA is safely used for steel rebar in concrete under marine environments. 

At the end of 30 days of exposure, the alkalinity of the O-SSA0, O-SSA10, O-SSA20, O-SSA30, O-SSA40, and O-SSA50 cement extracts were measured and given in Table 6. The alkalinity of the cement extract solution was decreased due to the action with atmospheric CO_2_ as follows:

(14) $$\mathrm{CaO}+{\;\mathrm{CO}}_{2}\;\to {\mathrm{CaCO}}_{3}$$

The pH was greatly affected for 40% and 50% replacement levels. However, the measured pH values were 10.14, 10.85, 11.21, and 10.37 for 0%, 10%, 20%, and 30% levels respectively. Here again, it was proved that the alkalinity (pH 10.14 to 11.21) was not much affected up to the 30%.

### 3.3. Surface Characterization of Steel Rebars

**Figure 10:**
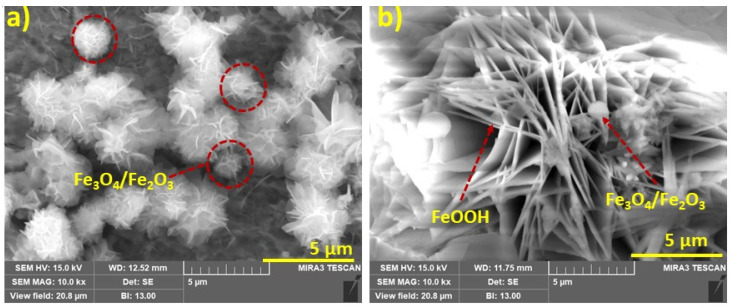
SEM micrographs of the corrosion products on the steel rebar surface after an exposure period of 30 days [O-SSSA0 (**a**); and O-SSA20 (**b**)].

Figure 10a,b illustrates the SEM microstructures of the steel rebar immersed in chloride contaminated O-SSA0 and O-SSA20, respectively after exposure periods of 30 days. Figure 10a shows the well-dispersed multiple flowers-like structures and randomly assembled with irregular shapes formed on the steel rebar surface. These flowers-like hierarchical structures correspond to the morphology of goethite (α-FeOOH) or α-Fe_2_O_3_/γ-Fe_2_O_3_ which indicates severe corrosion occurs on the steel rebar surface [55]. Figure 10b shows SEM microstructures of the steel rebar immersed in chloride contaminated O-SSA20 at the exposure period of 30 days. It can be seen from Figure 10b that the predominant laminar structures formed on the steel rebar surface, which correspond to the microstructures of the iron hydroxide/γ-FeOOH (lepidocrocite) [55,56]. This is due to the presence of abundance of -OH ions released from CaO in SSA.

**Figure 11:**
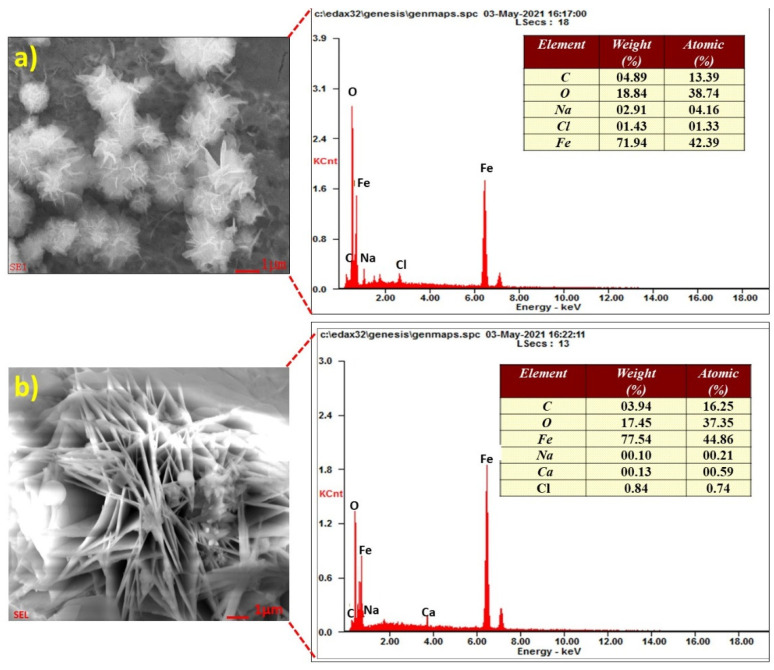
EDS analysis of the corrosion products on the steel rebar surface after an exposure period of 30 days [O-SSSA0 (**a**); and O-SSA20 (**b**)].

The EDS analysis after 30 days of the steel rebar immersed in chloride contaminated O-SSA0 and O-SSA20 are shown in Figure 11a,b. It was observed from Figure 11a,b that the major elements iron, oxygen, and chlorine were present on the steel rebar surface. From the elemental composition, the formation of iron oxide was established on the steel rebar surface.

**Figure 12:**
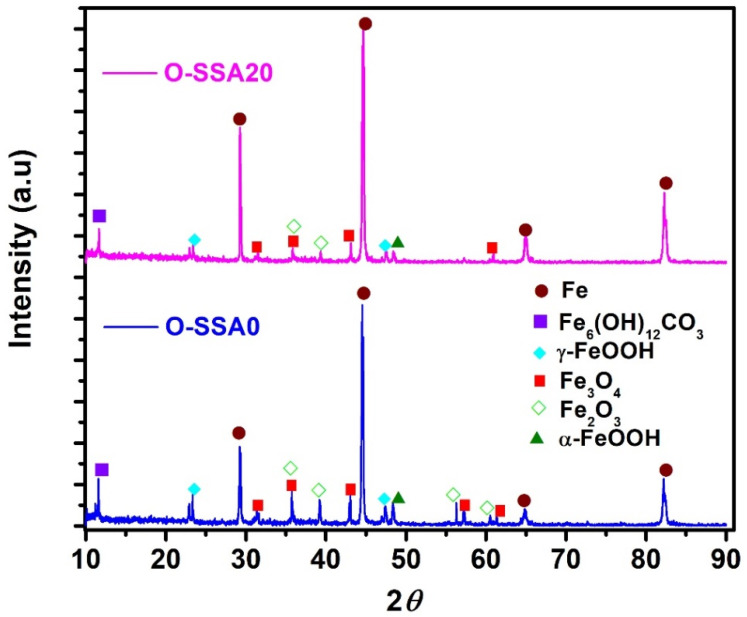
XRD patterns of the corrosion products on the steel rebar surface after an exposure period of 30 days [O-SSSA0 and O-SSA20].

Figure 12 shows the XRD pattern for corrosion products obtained on the steel rebar surface after the exposure period for 30 days for the systems O-SSA0 and O-SSA20, respectively. It can be seen from Figure 12 that the corrosion products were Fe_2_O_3_, α-FeOOH, γ-FeOOH (lepidocrocite), Fe_3_O_4_, and Fe_6_(OH)_12_CO_3_ [57,58,59,60]. In addition, the diffraction intensity of the Fe_3_O_4_/Fe_2_O_3_ is higher for the steel rebar immersed in a control system (O-SSA0) when compared to O-SSA20.

### 3.4. Mechanical Properties of Concrete

#### 3.4.1. Compressive Strength Test

**Figure 13:**
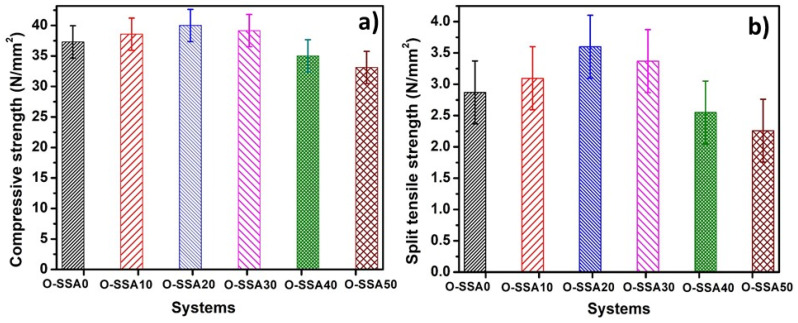
Mechanical properties of the concrete with a different replacement level of SSA. Compressive strength test (**a**); and split tensile strength test (**b**).

The average compressive strength vs. replacement level for control and OPC replaced by a SSA system at 10%, 20%, 30%, 40%, and 50% replacement levels are shown in Figure 13a.

It was interesting to note that the comparable compressive strength with OPC was observed up to 40% replacement levels. For example, the compressive strength for OPC (control) at 28 days was found to be 37.3 N/mm^2^ whereas the compressive strength for the system OPC + 40% SSA was found to be 35.03 N/mm^2^. However, the maximum compressive strength was noted for the system OPC + 20% SSA was found to be 40 N/mm^2^. There was a 1.07-time increase in compressive strength was observed at the 20% replacement level. A decrease in compressive strength was observed at the 50% replacement level. The compressive strength value was found to be 33.12 N/mm^2^ for the system OPC + 50% SSA. These data clearly illustrate that SSA blending well improved the strength of concrete. Here, it was worthwhile to mention that up to 30% of SSA can be safely replaced with the OPC in concrete constructions.

#### 3.4.2. Split Tensile Strength Test

Figure 13b shows the split tensile strength of O-SSA0, O-SSA10, O-SSA20, O-SSA30, O-SSA40, and O-SSA50, after 28 days cured samples. The split tensile strength for O-SSA0, O-SSA10, O-SSA20, O-SSA30, O-SSA40, and O-SSA50 were found to be 2.87, 3.09, 3.6, 3.37, 2.55, and 2.26 N/mm^2^, respectively. It was observed that the split tensile strength of O-SSA20 concrete showed higher split tensile strength than the O-SSA0. However, it was observed that the comparable split tensile strength with OPC was noted up to 40% replacement levels. For example, the split tensile strength for O-SSA0 (without SSA) at 28 days was found to be 2.87 N/mm^2^ whereas the split tensile strength for the system O-SSA40 (OPC + 40% SSA) was found to be 2.55 N/mm^2^. The gain in strength in SSA blended concrete is because the presence of CaO in SSA readily reacts with water to form Ca(OH)_2_ and further reacts with silica and alumina to form C-S-H gel and C-A-S-H. The cement hydration reactions are described below [61].

(15) $$\mathrm{SAA}\left(3\mathrm{Cao}\right)+3H_{2}O\to 3\mathrm{Ca}{\left(\mathrm{OH}\right)}_{2}$$

(16) $$\mathrm{SSA}\left(4\mathrm{CaO}\right)+2{\mathrm{SiO}}_{2}+4H_{2}O\to 3\mathrm{CaO}\cdot 2{\mathrm{SiO}}_{2}\cdot 3H_{2}O\;\left(C–S–H\right)+Ca{\left(OH\right)}_{2}$$

(17) $$\mathrm{SSA}\left(4\mathrm{CaO}\right)+2{\mathrm{SiO}}_{2}+{\mathrm{Al}}_{2}O_{3}+4H_{2}O\to 3\mathrm{Cao}\cdot Al_{2}O_{3}\cdot 2SiO_{2}\cdot 3H_{2}O\left(C–A–S–H\right)+Ca{\left(OH\right)}_{2}$$

The formation of C-S-H and C-A-S-H can improve the strength and durability of concrete [62]. It is a fact that SSA possesses a small mean particle size (90–150 μm), it permits Ca^2+^ ions which can diffuse internally, and therefore hydration of cement and pozzolanic reactions are continued for a long time.

### 3.5. Extrapolation Modeling

#### 3.5.1. Extrapolation Modeling for Compressive Strength with Later Age

**Figure 14:**
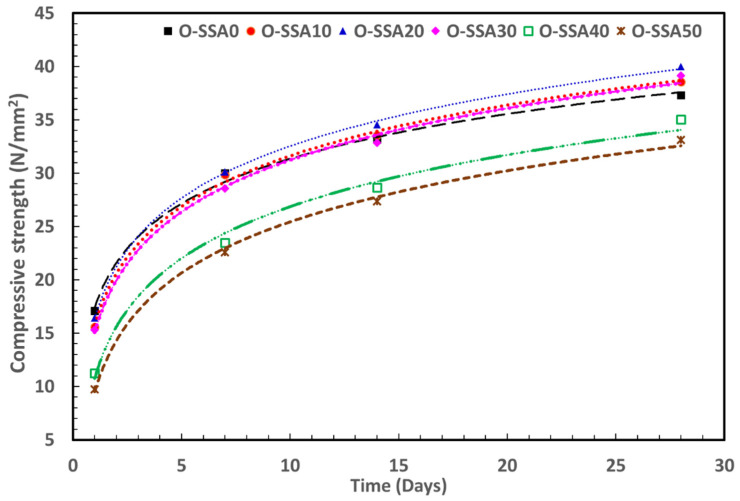
Compressive strength data vs. time (days).

The compressive strength of concrete measured at the different curing periods for extrapolation modeling is given in Table S1 (see supporting information). The compressive strength data is plotted using MS Excel with respect to curing days, as shown in Figure 14 to understand the pattern of curing. The relation of gain in compressive strength in later days (N) is found to be of logarithmic form [A*ln(N) + B] obtained after fitting data points in MS Excel. The values of A, B, and R^2^ are given in Table 7. Values for 3rd curing day are omitted for better fitting. The fitted equations are used to extrapolate compressive strength up to 365 exposure days (Table 7). The obtained values suggest that the 20% replacement of SSA will give a compressive strength of ~57.8 N/mm^2^ after 365 days under ideal conditions followed by 30% replacement of SSA, which gives compressive strength of ~56.5 N/mm^2^. Both these values are better than the control system (~53.2 N/mm^2^) and suggest that SSA can be used as a promising admixture in order to decrease the usage of cement in construction.

An attempt is made to obtain a curing rate in terms of change in compressive strength with the help of a difference method. In this method, compressive strength value is subtracted by its next value and % change is obtained. The formula can be written as:

(18) $$Change\;in\;compressive\;strength\;\left(\%\right)=\left[\frac{CS_{n+1}-CS_{n}}{CS_{n}}\right]\times 100$$

where *CS* = compressive strength and *n* = number of day.

The dataset is extrapolated up to 700 days (Figure S3 and Table S2 in the Supporting Information). It was found that the change in compressive strength start decreasing with time and falls below one percent after 150 days.

#### 3.5.2. Extrapolation Modelling for Corrosion Rate of Steel Rebar in Chloride Contaminated Various Cement Extracts

**Figure 15:**
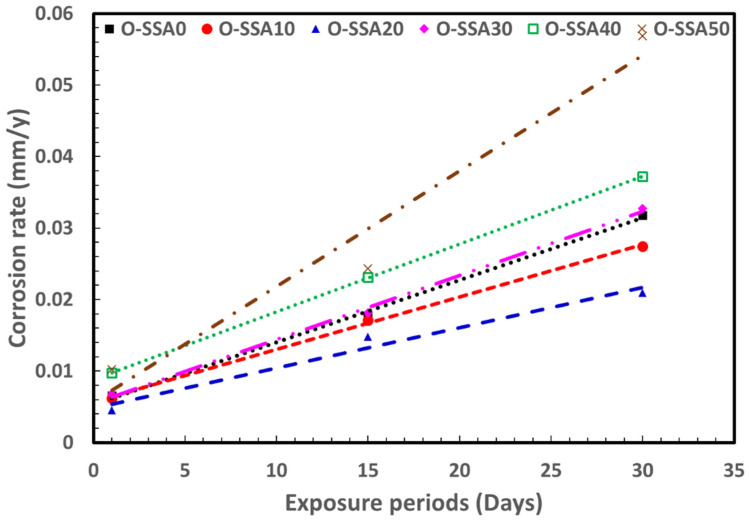
Corrosion rate vs. exposure periods.

The corrosion rate calculated using electrochemical method is plotted with respect to exposure days is given in Figure 15. Here, 20% SSA blending was found to give the best corrosion resistance. It was also observed that all the systems follow a linear pattern and relation in the corrosion rate and number of exposure days (NE) can be written as equation, A*NE +B. The data is fitted to linear equation using MS Excel and the equation is used to extrapolate a corrosion rate after 365 days of exposure. The comparison for various compositions is given in Table S3 in the supporting information. The obtained values suggest that 20% SSA blending will have a corrosion rate of 0.2238 mm/y after 365 days as compared to control with 0.3338 mm/y. The 20% SSA blending gives more than 30% improvement in corrosion resistance as compared to cement. Thus, SSA is recommended as promising candidate material to protect the steel rebars in marine environments.

## 4. Conclusions

The following broad conclusions can be drawn from the present investigations:

- Chrono-potential studies showed that the optimum cement replacement level of up to 20% of SSA is sufficiently suitable for better durability of the reinforced concrete structures under marine environments.
- Potentiodynamic polarization studies revealed that the comparable corrosion rate was obtained up to a 20% replacement level. At 20% replacement level of SSA (O-SSA20) showed lesser corrosion current density (0.3857, 1.2536, and 1.7815 μA/cm^2^) and corrosion rate values (0.4539, 1.4755, and 2.0969 mm/y) at all the exposure periods (1, 15, and 30 days), which compared to all other systems.
- Weight loss measurements indicated that there was a reduction in the corrosion rate of rebar by 1.408 times at a 20% replacement level and 1.157 times at a 10% replacement level when compared to control. The quantitative data confirmed that up to a critical level of 20% to 30% replacement, SSA improved the corrosion resistance of concrete.
- Mechanical properties of SSA blended concrete, such as compressive strength and split tensile strength showed that up to a 30% replacement level, SSA improved the strength of concrete.
- Comparable corrosion rate with control was noticed up to a 30% replacement level. Hence, the cement replacement level of 30% by SSA is safely used for steel rebar in concrete under marine environments.
- EIS studies revealed that at a 20% replacement level, a gradual increase of film resistance across the steel-concrete interfacial regions was noticed. At higher replacement levels, chloride ions penetrate the iron hydroxide layer on the steel rear surface and it has become diffused/porous, thus the speed of corrosion rate on the steel rebar surface.
- Surface examinations inferred that the formation of iron oxide and iron hydroxide rust products on the steel rebar under aggressive marine environments.
- It was concluded that the animal origin from the marine was found to be a sustainable engineering material for construction industries and also for the marine environment. SSA is a suitable replacement material for natural limestone in cement productions.
- The fitting equations are used to extrapolate compressive strength up to 365 curing days. The obtained values suggest that the 20% replacement of SSA will give a compressive strength of ~57.8 N/mm^2^ after 365 days of curing under ideal conditions followed by 30% replacement of SSA, which gives compressive strength of ~56.5 N/mm^2^.

## Figures and Tables

**Table 1:** Chemical composition of ordinary Portland cement (OPC) and Thermomechanical treated (TMT) steel rebar.

OPC		Steel Rebar	
Constituents	wt (%)	Constituents	wt (%)
CaO	63.41	Iron (Fe)	98.498
SiO_2_	21.96	Carbon (C)	0.236
Al_2_O_3_	5.27	Silica (Si)	0.249
Fe_2_O_3_	3.44	Chromium (Cr)	0.037
MgO	2.13	Nickel (Ni)	0.028
Na_2_O	0.12	Manganese (Mn)	0.90
K_2_O	0.43	Molybdenum (Mo)	0.009
SO_3_	2.45	Copper (Cu)	0.018
LOI	0.79	Phosphorous (P)	0.014
		Tin (Sn)	0.002
		Sulphur (S)	0.009
Physical properties			
Specific gravity (g/cm^3^)	3.15	Density (g/cm^3^)	7.85

**Table 2:** Formulation of chloride contaminated various cement extracts.

System	Cement (g)	SSA (g)	Distilled Water (mL)	NaCl (g)	pH
O-SSA0	100	0	100	3.5	12.73
O-SSA10	90	10			12.75
O-SSA20	80	20			12.78
O-SSA30	70	30			12.76
O-SSA40	60	40			12.77
O-SSA50	50	50			12.76

**Table 3:** Mix proportions of concrete with SSA.

System	Cement (kg/m^3^)	SSA (kg/m^3^)	Fine Aggregates (kg/m^3^)	Coarse Aggregates (kg/m^3^)	Water/Cement Ratio
O-SSA0	415.0	0	710	1287	0.5
O-SSA10	373.5	41.5	710	1287	
O-SSA20	332.0	83.0	710	1287	
O-SSA30	290.5	124.5	710	1287	
O-SSA40	249.0	166.0	710	1287	
O-SSA50	207.5	207.5	710	1287	

**Table 4:** Corrosion kinetic parameters for steel rebars in various cement extracts for a various exposure period obtained from Tafel plots.

System	Exposure Period (Days)	E_corr_ (mV) vs. SCE	ba (mV/dec)	bc (mV/dec)	I_corr_ (µA/cm^2^)	Corrosion Rate (mm/y) ×10^−2^
O-SSA0 (OPC)	1	−611	114	102	0.5572	0.6558
	15	−680	124	369	1.5062	1.7728
	30	−751	76	161	2.6981	3.1757
O-SSA10	1	−597	115	131	0.5239	0.6167
	15	−677	112	364	1.4549	1.7125
	30	−731	137	127	2.3321	2.7449
O-SSA20	1	−593	104	117	0.3857	0.4539
	15	−668	111	272	1.2536	1.4755
	30	−706	103	168	1.7815	2.0969
O-SSA30	1	−619	72	223	0.5736	0.6751
	15	−690	111	311	1.5382	1.8105
	30	−753	116	110	2.6915	3.1679
O-SSA40	1	−629	145	149	0.8255	0.9716
	15	−698	124	395	1.9648	2.3126
	30	−762	128	100	3.1637	3.723
O-SSA50	1	−660	130	125	0.8719	1.0263
	15	−702	145	482	2.0630	2.4281
	30	−785	195	122	4.8319	5.6873

**Table 5:** Impedance parameters for steel rebars in various cement extracts for various exposure periods obtained from Nyquist plots.

System	Exposure Period (Days)	R_S_ (Ω·cm^2^)	R_1_ (Ω·cm^2^)	CPE_1_		R_ct_	CPE_ct_	
				Y0 (Ω^−1^·cm^2^ S^−n^) × 10^−3^	N	(Ω·cm^2^)	Y0 (Ω^−1^·cm^2^ S^−n^) ×10^−2^	N
O-SSA0 (OPC)	1	1.58	–	–	–	2097	0.131	0.74
	15	1.99	969.6	1.598	0.66	1063	1.082	0.77
	30	2.85	157.9	7.307	0.63	176	1.942	0.74
O-SSA10	1	2.201	–	–	–	2201	0.125	0.75
	15	1.59	975.8	2.148	0.65	1142	1.064	0.83
	30	3.03	180.7	3.061	0.62	289	1.224	0.70
O-SSA20	1	1.335	–	–	–	2281	0.123	0.74
	15	2.48	989.8	1.523	0.65	1239	0.929	0.76
	30	2.53	243.9	2.606	0.65	385	1.150	0.72
O-SSA30	1	1.414	–	–	–	1924	0.134	0.74
	15	2.04	966.9	1.924	0.60	971	1.115	0.76
	30	3.17	159.5	6.6344	0.72	183	1.313	0.84
O-SSA40	1	0.955	–	–	–	1875	0.161	0.70
	15	2.32	946.7	1.527	0.69	894	1.353	0.86
	30	2.39	157.1	8.007	0.69	175	2.015	0.68
O-SSA50	1	2.61	–	–	–	1410	0.164	0.73
	15	2.81	920.2	1.792	0.67	799	1.966	0.92
	30	2.64	100.8	5.894	0.68	158	2.835	0.63

**Table 6:** Corrosion rate for steel rebars in various cement extracts obtained from gravimetric weight loss measurements.

System	Number of Specimens	Weight Loss (g)	Corrosion Rate (mm/y)	Average Corrosion Rate (mm/y)	pH	
					Initial	Final
O-SSA0 (OPC)	1	0.7753	0.7973	0.7998	12.73	10.14
	2	0.7801	0.8022		12.75	10.43
O-SSA10	1	0.6746	0.6937	0.6913	12.75	10.85
	2	0.6698	0.6888		12.74	10.29
O-SSA20	1	0.5499	0.5655	0.5679	12.78	11.21
	2	0.5546	0.5703		12.76	11.35
O-SSA30	1	0.7747	0.7966	0.7983	12.76	10.37
	2	0.7779	0.7999		12.77	10.41
O-SSA40	1	0.8301	0.8536	0.8586	12.77	10.08
	2	0.8398	0.8636		12.77	10.12
O-SSA50	1	0.8662	0.8907	0.8869	12.76	9.92
	2	0.8589	0.8832		12.77	10.07

**Table 7:** Values of A, B, R^2^, and compressive strength at the 365th day with respect to a different replacement level.

Replacement Level (%)	A	B	R^2^	Compressive Strength at 365th Day (N/mm^2^)
O-SSA0	6.0585	17.403	0.9964	53.15
O-SSA10	6.8661	15.818	0.9977	56.33
O-SSA20	7.0229	16.372	0.9994	57.81
O-SSA30	7.0118	15.092	0.9963	56.46
O-SSA40	6.9946	10.763	0.9926	52.03
O-SSA50	6.9208	9.5042	0.9977	50.33

## Data Availability

The raw/processed data required to reproduce these findings cannot be shared at this time as the data also forms part of an ongoing study.

## Supplementary Materials

The following are available online at https://www.mdpi.com/article/10.3390/ma14237286/s1, Figure S1: Equivalent circuit model fit for EIS analysis of the steel rebar immersed in chloride contaminated 30 various cement extracts for an exposure period of 1 day (a); 15 days and 30 days (b); Figure S2: Photographic image of steel rebars immersed in chloride contaminated various cement extracts 35 after exposure periods of 30 days; Figure S3: Change in compressive strength vs. Time (Days) (Extrapolation modelling); Table S1: Compressive strength at different curing periods (days); Table S2: Change in compressive strength vs. Time (days); Table S3: Value of A, B, R2 and estimated corrosion rate from linear extrapolation (Equation: A * NE + B).
