# Supplementary material for: Assessment of Corrosion Performance of Steel Rebar in Snail Shell Ash Blended Cements under Marine Environments
Source: Materials (Basel). 2021 Nov 28;14(23):7286. doi: 10.3390/ma14237286 (PMC8658291; doi:10.3390/ma14237286)
Supplement: Supplementary file 1 [file materials-14-07286-s001.zip › materials-1460359-supplementary.pdf]

materials

Supplementary Materials

Assessment of Corrosion Performance of Steel Rebar in SSA Blended Cements under Marine Environments

Raghav Muralidharan [1], Karthick Subbiah [2*], Taejoon Park[3] and Han-Seung Lee[4*]

[1] Department of Civil Engineering, PSG Institute of Technology and Applied Research, Neelambur, Coimbatore 641 062, India; raghav09psgitech@gmail.com
[2] Department of Architectural Engineering, Hanyang University, , 55 Hanyangdaehak-ro, Sangrok-gu, Gyeonggi-do, Ansan-si 15588, Korea
[3] Department of Robotics Engineering, Hanyang University, 55 Hanyangdaehak-ro, Gyeonggi-do, Ansan-si 15588, Korea; taejoon@hanyang.ac.kr

* Correspondence: karthick.chemistry@gmail.com (K.S.); ercleehs@hanyang.ac.kr (H.-S.L.); Tel.: +82-31-436-8159 (H.-S.L.); Fax: +82-31-436-8169 (H.-S.L.)

Citation: Raghav, M.; Karthick, S.; Park, T.; Lee, H.-S. Assessment of Corrosion Performance of Steel Rebar in SSA Blended Cements under Marine Environments. *Materials* **2021**, *14*, 7286. https://doi.org/10.3390/ma14237286

Academic Editor: Frank Collins

Received: 28 October 2021
Accepted: 24 November 2021
Published: 28 November 2021

Publisher's Note: MDPI stays neutral with regard to jurisdictional claims in published maps and institutional affiliations.

Copyright: © 2021 by the authors. Licensee MDPI, Basel, Switzerland. This article is an open access article distributed under the terms and conditions of the Creative Commons Attribution (CC BY) license (http://creativecommons.org/licenses/by/4.0/).

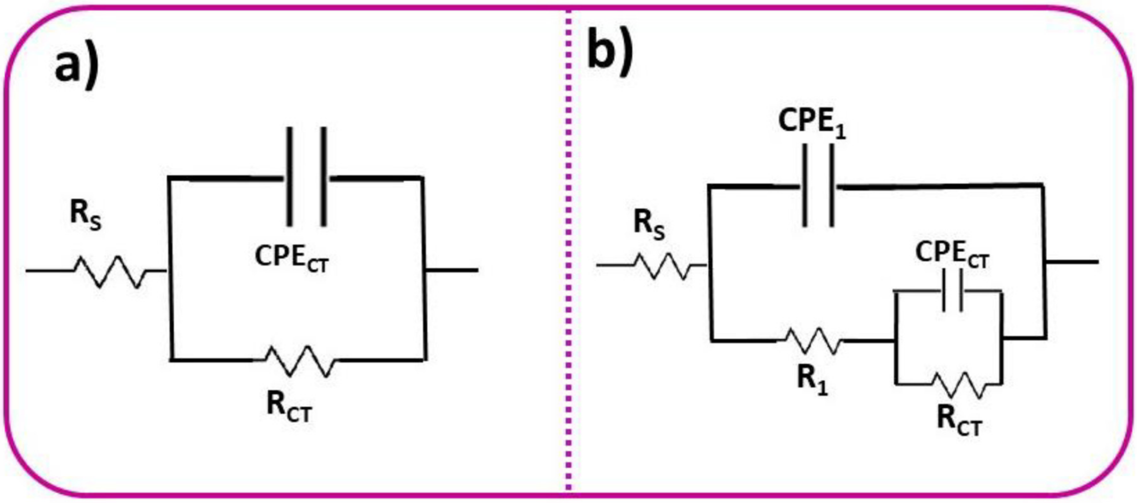

Figure S1. Equivalent circuit model fit for EIS analysis of the steel rebar immersed in chloride contaminated 30 various cement extracts for an exposure period of 1 day (**a**); 15 days and 30 days (**b**).

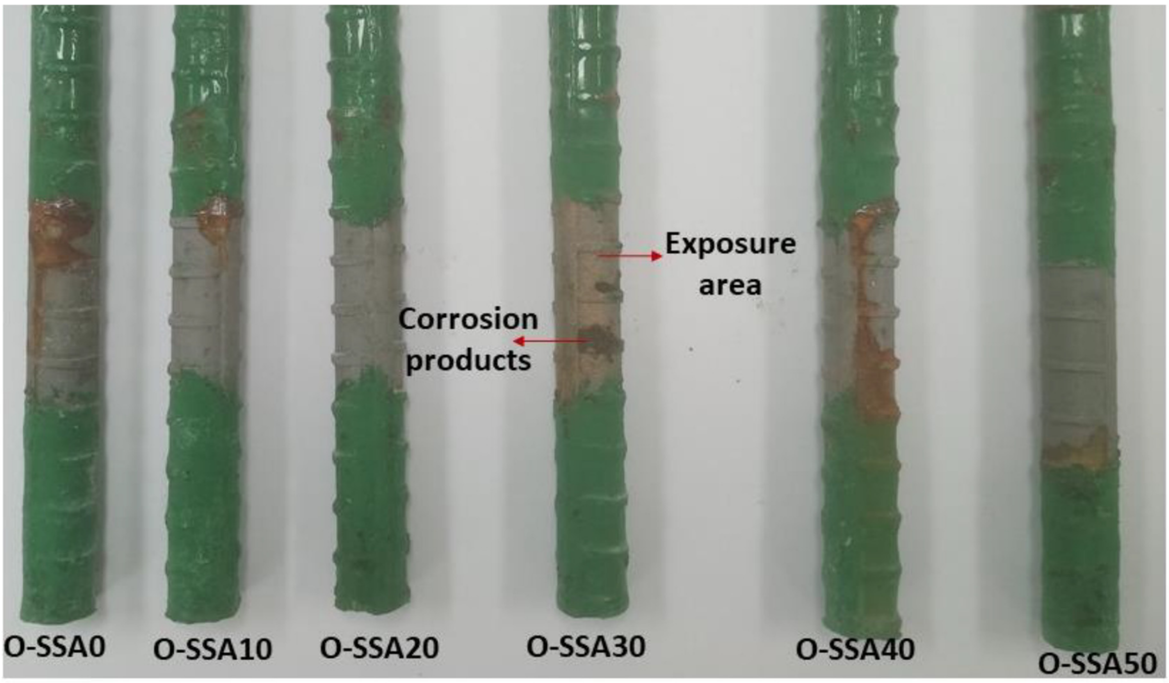

Figure S2. Photographic image of steel rebars immersed in chloride contaminated various cement extracts 35 after exposure periods of 30 days.

Table S1. Compressive strength at different curing periods (days).

	Compressive Strength with Different Curing Days (N/mm^2)				
System	1 day	3 days	7 days	14 days	28 days
O-SSA0	17.12	20.74	29.98	33.18	37.30
O-SSA10	15.60	19.25	29.88	33.58	38.57
O-SSA20	16.41	20.15	30.12	34.56	40.00
O-SSA30	15.32	18.78	28.56	32.85	39.15
O-SSA40	11.25	13.86	23.48	28.67	35.03
O-SSA50	9.75	12.55	22.59	27.35	33.12

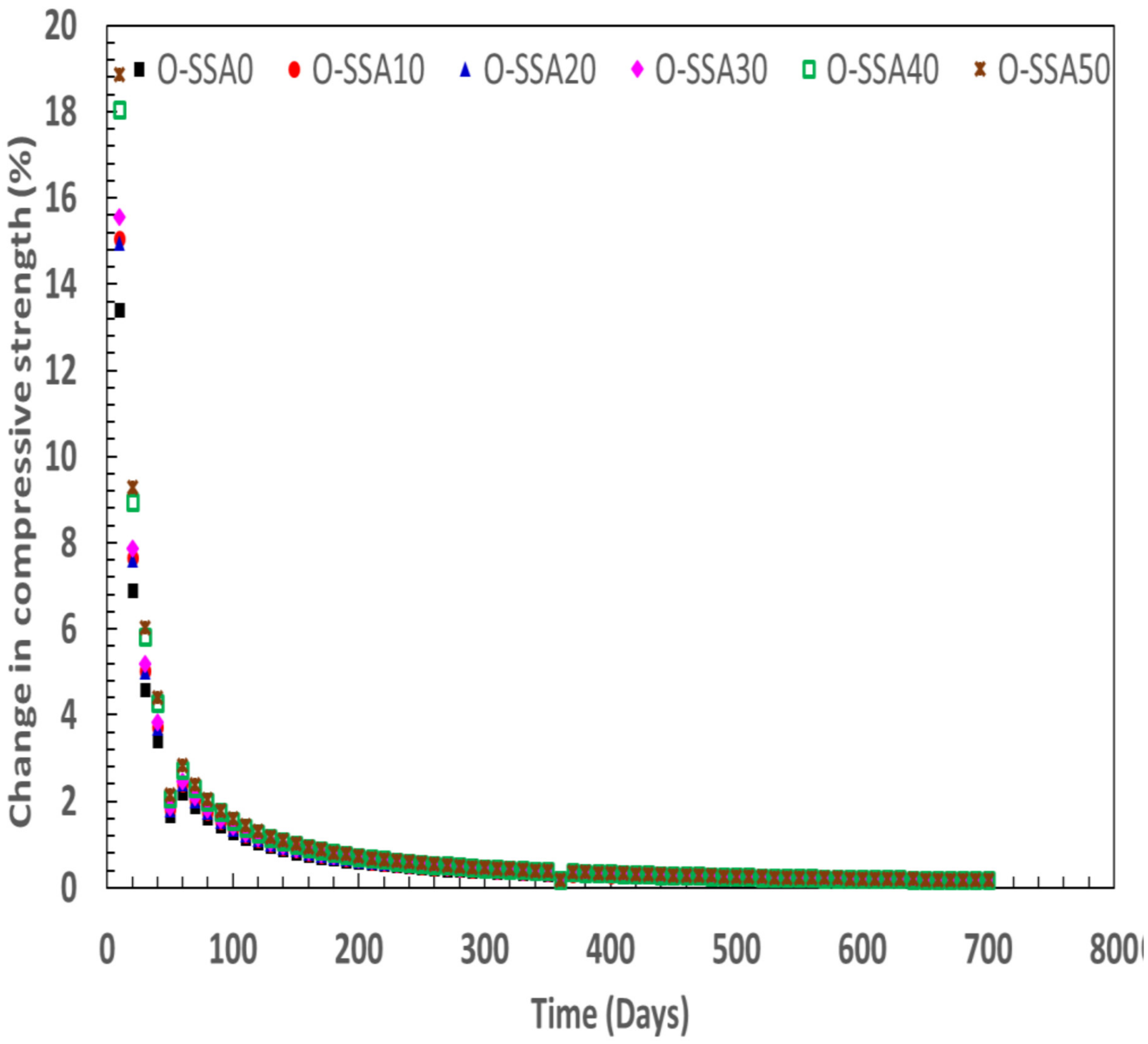

Figure S3. Change in compressive strength vs. Time (Days) (Extrapolation modelling).

Table S2. Change in compressive strength vs. Time (days).

	Change in Compressive Strength (%)					
Days	O-SSA0	O-SSA10	O-SSA20	O-SSA30	O-SSA40	O-SSA50
10	13.39395	15.04759	14.95845	15.55901	18.04439	18.85671
20	6.909501	7.650986	7.611562	7.876009	8.9418	9.280474
30	4.585532	5.042648	5.018503	5.18013	5.823576	6.025414
40	3.400867	3.723613	3.706636	3.820133	4.268535	4.40807
50	1.670402	1.823234	1.815218	1.868755	2.079127	2.144223
60	2.212635	2.40931	2.399018	2.467707	2.736514	2.819335
70	1.875181	2.037939	2.029437	2.086145	2.307336	2.375253
80	1.623581	1.761687	1.754484	1.802508	1.989314	2.046512
90	1.42914	1.548601	1.542379	1.583849	1.744793	1.793954
100	1.274598	1.379517	1.374058	1.410427	1.55129	1.594229
110	1.148972	1.242263	1.237414	1.26971	1.394583	1.432578
120	1.044946	1.128749	1.124397	1.153376	1.265247	1.299232
130	0.957464	1.033394	1.029454	1.055683	1.1568	1.187473
140	0.882924	0.952227	0.948633	0.972551	1.064639	1.092537
150	0.818692	0.882347	0.879048	0.900998	0.985412	1.010955

160	0.762798	0.821588	0.818543	0.8388	0.91662	0.940142
170	0.71374	0.768302	0.765477	0.784263	0.856363	0.878134
180	0.670356	0.72121	0.718579	0.736076	0.803172	0.823413
190	0.631728	0.679309	0.676848	0.693209	0.755894	0.774788
200	0.597128	0.641799	0.63949	0.654841	0.713612	0.731312
210	0.565964	0.608034	0.60586	0.62031	0.675587	0.692222
220	0.537758	0.57749	0.575438	0.589077	0.641219	0.656899
230	0.512114	0.549734	0.547791	0.560699	0.610013	0.624833
240	0.488703	0.524406	0.522564	0.534809	0.58156	0.595601
250	0.46725	0.501208	0.499456	0.511097	0.555517	0.56885
260	0.447522	0.479884	0.478215	0.489305	0.531596	0.544282
270	0.429324	0.460222	0.458628	0.469212	0.509552	0.521647
280	0.412486	0.442036	0.440513	0.45063	0.489177	0.500727
290	0.396863	0.425169	0.423711	0.433399	0.470291	0.48134
300	0.382332	0.409486	0.408087	0.417378	0.452739	0.463325
310	0.368782	0.394867	0.393523	0.402445	0.436388	0.446545
320	0.35612	0.38121	0.379918	0.388497	0.421121	0.430879
330	0.344262	0.368424	0.367181	0.37544	0.406835	0.416222
340	0.333135	0.356431	0.355232	0.363192	0.393441	0.40248
350	0.322675	0.345159	0.344002	0.351683	0.380858	0.389573
360	0.157483	0.168419	0.167856	0.171591	0.185772	0.190007
370	0.303531	0.324538	0.323458	0.330631	0.357854	0.365981
380	0.294752	0.315085	0.31404	0.320981	0.347316	0.355174
390	0.286445	0.306143	0.30513	0.311853	0.33735	0.344956
400	0.278573	0.297672	0.29669	0.303206	0.327913	0.335281
410	0.271105	0.289636	0.288684	0.295005	0.318965	0.326108
420	0.264009	0.282004	0.281079	0.287216	0.310469	0.317399
430	0.257261	0.274746	0.273847	0.279809	0.302392	0.309121
440	0.250834	0.267835	0.266962	0.272757	0.294705	0.301242
450	0.244707	0.261248	0.260399	0.266036	0.287381	0.293736
460	0.238859	0.254964	0.254137	0.259624	0.280394	0.286577
470	0.233273	0.248961	0.248155	0.2535	0.273723	0.279742
480	0.227931	0.243222	0.242437	0.247645	0.267347	0.273209
490	0.222818	0.23773	0.236964	0.242042	0.261248	0.26696
500	0.21792	0.232469	0.231722	0.236676	0.255407	0.260977
510	0.213224	0.227426	0.226697	0.231532	0.249809	0.255243
520	0.208717	0.222588	0.221876	0.226597	0.24444	0.249743
530	0.204389	0.217942	0.217247	0.221859	0.239286	0.244464
540	0.200229	0.213477	0.212798	0.217305	0.234334	0.239393
550	0.196228	0.209184	0.20852	0.212927	0.229574	0.234518
560	0.192378	0.205053	0.204403	0.208714	0.224994	0.229828
570	0.188669	0.201074	0.200438	0.204657	0.220584	0.225313
580	0.185094	0.19724	0.196617	0.200747	0.216336	0.220964
590	0.181647	0.193543	0.192933	0.196978	0.212241	0.216771
600	0.178321	0.189976	0.189379	0.193341	0.208291	0.212727
610	0.175109	0.186533	0.185947	0.18983	0.204478	0.208823
620	0.172006	0.183206	0.182632	0.186439	0.200795	0.205054
630	0.169006	0.179991	0.179429	0.183161	0.197237	0.201411
640	0.166105	0.176882	0.17633	0.179992	0.193797	0.19789
650	0.163298	0.173874	0.173332	0.176925	0.190469	0.194484
660	0.16058	0.170962	0.17043	0.173957	0.187248	0.191187

670	0.157947	0.168142	0.16762	0.171082	0.184129	0.187995
680	0.155396	0.165409	0.164896	0.168296	0.181107	0.184903
690	0.152922	0.16276	0.162256	0.165596	0.178179	0.181906
700	0.150523	0.16019	0.159695	0.162977	0.175339	0.179

Table S3. Value of A, B, R2 and estimated corrosion rate from linear extrapolation (Equation: A*NE + B).

% of Replacement	A	B	R2	Corrosion Rate (mm/y) after 365 Days Exposure
O-SSA0	0.0009	0.0053	0.9979	0.3338
O-SSA10	0.0007	0.0057	0.9986	0.2612
O-SSA20	0.0006	0.0048	0.9747	0.2238
O-SSA30	0.0009	0.0054	0.9973	0.3339
O-SSA40	0.0009	0.0088	1.0000	0.3373
O-SSA50	0.0016	0.0057	0.9583	0.5897

3.6. General Cost Estimation

Let us take 1 bag cement is 50 kg 61

If the replacement levels increase the consumption of cement in the formulation decreases as follows:

O-SSA10 (10% × 50) = 5 kg

O-SSA20 (20% × 50) = 10 kg

O-SSA30 (30% × 50) = 15 kg

1 ton of cement ≈ 50 USD

O-SSA10 (10% × 50) = 5 USD

O-SSA20 (20% × 50) = 10 USD

O-SSA30 (30% × 50) = 15 USD

SSA in concrete will save cost of 15 USD for the use of 1 ton of cement.
